# INdividual Vocational and Educational Support Trial (INVEST) for young people with borderline personality disorder: study protocol for a randomised controlled trial

**DOI:** 10.1186/s13063-020-04471-3

**Published:** 2020-06-26

**Authors:** Andrew M. Chanen, Katie Nicol, Jennifer K. Betts, Gary R. Bond, Cathrine Mihalopoulos, Henry J. Jackson, Katherine N. Thompson, Martina Jovev, Hok Pan Yuen, Gina Chinnery, Judith Ring, Kelly Allott, Louise McCutcheon, Ashleigh P. Salmon, Eoin Killackey

**Affiliations:** 1 Orygen, 35 Poplar Road, Parkville, Melbourne, VIC 3052 Australia; 2 Centre for Youth Mental Health, The University of Melbourne, 35 Poplar Road, Parkville, Melbourne, VIC 3052 Australia; 3 IPS Employment Center, Rockville Institute and Westat Inc., 85 Mechanic Street, Suite C3-1, Box 4A, Lebanon, NH 03766 USA; 4 Deakin Health Economics, Centre for Population Health Research, Deakin University, Geelong, VIC 3220 Australia; 5 Melbourne School of Psychological Sciences, Redmond Barry Building, The University of Melbourne, Parkville, Melbourne, VIC 3010 Australia; 6 Travancore School, 35 Poplar Road, Parkville, Melbourne, VIC 3052 Australia

**Keywords:** Individual placement and support, Borderline personality disorder, Youth, Early intervention, Adolescents, Young adults, Education, Employment

## Abstract

**Background:**

The clinical onset of borderline personality disorder (BPD) usually occurs in young people (aged 12–25 years) and commonly leads to difficulty achieving and maintaining vocational (education and/or employment) engagement. While current psychosocial interventions lead to improvements in psychopathology, they have little effect upon functioning. Individual Placement and Support (IPS) is a client-driven model that assists individuals with severe mental illness to engage with education and/or employment appropriate to their personal goals, and that provides ongoing support to maintain this engagement. The objective of the INdividual Vocational and Educational Support Trial (INVEST) is to evaluate the effectiveness of adding IPS to an evidence-based early intervention programme for BPD, with the aim of improving vocational outcomes.

**Methods/design:**

INVEST is a single-blind, parallel-groups, randomised controlled trial (RCT). The randomisation is stratified by gender and age and uses random permuted blocks. The interventions are 39 weeks of either IPS, or ‘usual vocational services’ (UVS). Participants will comprise 108 help-seeking young people (aged 15–25 years) with three or more *DSM-5* BPD features and a desire to study or work, recruited from the Helping Young People Early (HYPE) early intervention programme for BPD at Orygen, in Melbourne, Australia. All participants will receive the HYPE intervention. After baseline assessment, staff who are blind to the intervention group allocation will conduct assessments at 13, 26, 39 and 52 weeks. At the 52-week primary endpoint, the primary outcome is the number of days in mainstream education/employment since baseline. Secondary outcomes include the cost-effectiveness of the intervention, quality of life, and BPD severity.

**Discussion:**

Current treatments for BPD have little impact on vocational outcomes and enduring functional impairment is prevalent among this patient group. IPS is a targeted functional intervention, which has proven effective in improving vocational outcomes for adults and young people with psychotic disorders. This trial will investigate whether IPS is effective for improving vocational (employment and educational) outcomes among young people with subthreshold or full-syndrome BPD.

**Trial registration:**

Australian New Zealand Clinical Trials Registry, ID: ACTRN12619001220156. 13 September 2019.

## Background

Borderline personality disorder (BPD) is a severe mental disorder that is characterised by difficulty managing interpersonal relationships, an unstable sense of self, intense and volatile emotions and impulsive behaviours [1]. The clinical onset of BPD usually occurs between puberty and young adulthood (‘young people’; aged 12–25 years) [2]. From this age, BPD is associated with substantial and enduring impairments in social and occupational functioning [3–6]. While these impairments are associated with characteristics of BPD, such as impulsivity [7], prospective studies have consistently found that functional impairment persists long after the diagnostic features of the disorder have attenuated [8–11].

BPD in young people predicts the persistence of functional impairments into adulthood [5, 6]. The severity of BPD features at age 14 is associated with lower academic and occupational attainment 20 years later [6]. Personality disorder severity at age 24 years is associated with the absence of post-school qualifications, receipt of disability welfare payments, and relationship difficulties at age 35 years [5]. Young people, aged 15–18 years, who experience severe mental illness, including BPD, are three times more likely to be disengaged from education than their peers in the general population [12]. Failure to begin tertiary education is associated with being not in education, employment or training (NEET) among 19–25 year olds [13] and, in one study of young people at entry into a specialist BPD treatment programme, 62% were either partially or fully NEET [14].

The enduring effects of early vocational disruption in this group are evident, with BPD more strongly associated with unemployment and impairment at work than depression or anxiety [15]. BPD features are consistently linked with impaired work performance, with even the presence of subthreshold features (1–4 *Diagnostic and Statistical Manual of Mental Disorders, version 5* (*DSM-5*) criteria) being associated with impaired work performance in a population sample of 18–64 year-olds [16].

Among outpatients aged 15–25 years, subthreshold features of BPD were associated with poorer occupational functioning than among those with no BPD features [17]. Of all personality disorders, BPD is the most strongly associated with receipt of disability welfare payments [18, 19], and people with BPD are more than eight times more likely to be receiving disability welfare payments than individuals with no personality disorder [19]. Over a 10-year follow-along period, people with BPD were three times more likely to be receiving disability welfare payments than those with other personality disorders [20]. Although 40% of those BPD patients were able to cease reliance on disability welfare payments at some point, almost half of those who ceased subsequently resumed benefits [20]. At the 10-year assessment, only 55% of the study population reported having worked or attended school for at least half of the previous 2 years. Consequently, BPD is among the most costly mental disorders [21], with indirect (decreased or lost productivity), rather than direct (treatment-related), costs accounting for the majority of the costs attributable to BPD [22–24].

Despite wanting to work in the open labour market [25], people with BPD struggle to maintain employment, and they find the experience of working more stressful than individuals with other mental disorders [26]. While improvements in psychopathology are observed following psychological intervention [27], this does not translate into substantial or sustained improvements in functioning [8]. For example, BPD patients treated with 1 year of dialectical behavioural therapy (DBT) showed no improvement in occupational functioning following treatment, or at 1-year follow-up [24].

BPD patients identify employment as helpful for recovery [25]. Self-reported effects of employment among those experiencing mental ill-health include improved self-esteem and self-worth, societal acceptance, and increased optimism [28]. Further, employment is viewed as a benchmark of recovery in those experiencing mental illness [29], including among young people recently diagnosed with a severe mental disorder [30, 31]. Lack of employment can have adverse consequences upon well-being [32], exacerbating the stigmatisation so commonly experienced by individuals with BPD. Unemployment at a young age also has ‘scarring’ effects, making future unemployment more likely [33, 34]. Therefore, specialised and targeted vocational support appears to be necessary to optimise recovery among young people with BPD and to prevent long-term disability.

Individual Placement and Support (IPS) has been specifically designed to provide vocational support for people living with severe mental illness (i.e., psychotic disorders) [35]. It is a client-driven model, delivered by a vocational specialist, that allows for ongoing personalised support, even after a suitable placement has been obtained. IPS has consistently proved more effective than other employment services in randomised controlled trials (RCTs) in countries with varying labour markets, economic conditions and attitudes towards mental health [32, 36–42]. IPS, inclusive of educational support, has been implemented successfully in young people experiencing first-episode psychosis [43–46]. While IPS has been adapted for use in other mental disorders among young people [47], no RCT has investigated its effectiveness among young people with BPD.

### Aims and hypotheses

The overall purpose of the INdividual Vocational and Educational Support Trial (INVEST) is to evaluate the effectiveness of IPS for improving vocational (educational and/or occupational) outcomes for young people with either subthreshold (three to four features) or full-syndrome (five or more features) BPD, compared with routinely offered, usual vocational services (UVS) within a specialist BPD outpatient programme.

The primary hypothesis is that, at the 52-week primary endpoint, participants receiving IPS will have better outcomes on the primary (number of days in mainstream education/employment since baseline) and secondary (cost-effectiveness of the intervention, quality of life, BPD severity) outcome measures than those receiving UVS.

## Methods/design

### Study design

**Fig. 1:**
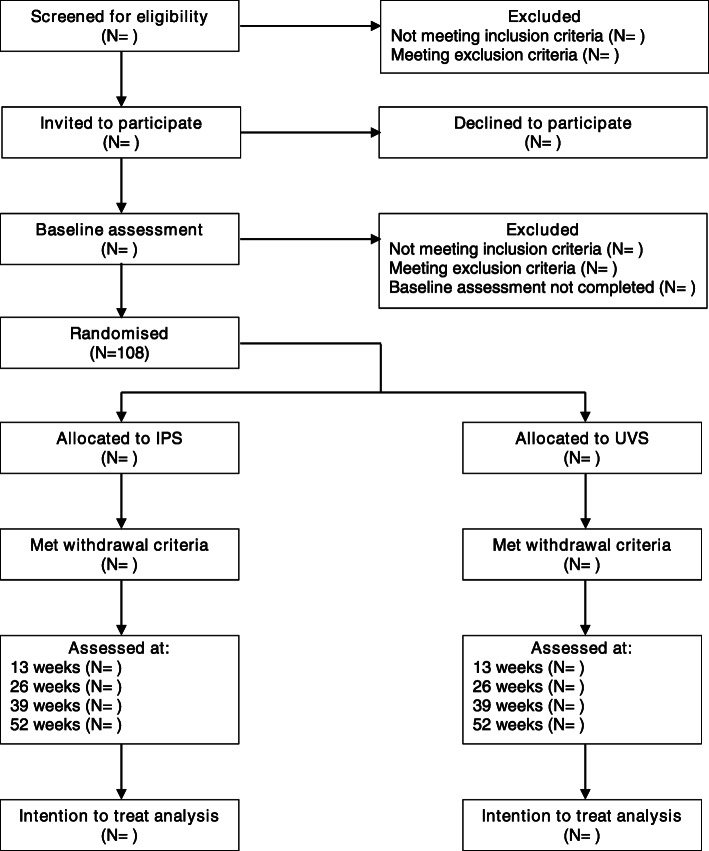
Consolidated Standards of Reporting Trias (CONSORT) flow diagram for the INdividual Vocational and Educational Support Trial (INVEST)

INVEST is a single-blind, parallel-groups RCT of IPS, compared with UVS. The study design was developed in accordance with Good Clinical Practice (GCP) Guidelines and Standard Protocol Items: Recommendations for Interventional Trials (SPIRIT) [48]. Figure 1 summarises the trial design. Proposed or necessary changes to the protocol will be submitted to Melbourne Health Human Research Ethics Committee for approval, and communicated to all relevant parties. The trial was prospectively registered with the Australian New Zealand Clinical Trial Registry (ACTRN12619001220156) and has been approved by the Melbourne Health Human Research Ethics Committee (HREC/18/MH/257). The trial is funded through a project grant awarded by the National Health and Medical Research Council, Australia (GNT1144022).

### Setting

All study participants will receive standard care in the Helping Young People Early (HYPE) clinical programme [49] at Orygen, Melbourne, Australia. Orygen is the Victorian State Government-funded specialist mental health service for 15- to 25-year-olds residing in western metropolitan Melbourne, Australia. HYPE integrates general psychiatric care, clinical case management, and time-limited, individual psychotherapy within an overall relational framework (Relational Clinical Care) that is derived from Cognitive Analytic Therapy (CAT) [50]. This treatment package is typically delivered over a period of 6–7 months. Patients are then offered up to four follow-up appointments over the subsequent 6 months, allowing adequate time to complete IPS or UVS.

HYPE provides a standardised background clinical treatment for all INVEST participants. HYPE adherence and competency are routinely monitored, at least weekly, through clinical supervision and clinical review processes. Individuals randomised to the IPS group will have contact with an IPS practitioner over the 39-week intervention period, in accordance with IPS principles.

The study will be conducted by Orygen and the Centre for Youth Mental Health, The University of Melbourne. Orygen, Melbourne, Australia is the study sponsor.

### Participants

Participants will be recruited from the HYPE programme at Orygen, Melbourne, Australia. Potential participants will be identified by case managers, referred to the research team and invited to participate in the study.

#### Inclusion criteria

- Young people (all genders) aged 15–25 years inclusive
- Three or more BPD features, assessed by the Structured Clinical Interview for *DSM-5* Personality Disorders (SCID 5-PD)
- A desire to enter mainstream education and/or employment (in which they are not currently engaged)
- Ability to give informed consent and adhere to study protocol
- Sufficient fluency in English

#### Exclusion criteria

- Participation interferes with the appropriate clinical management of risk of harm (or actual harm) to self or others
- Participation in another clinical trial which, in the opinion of the principal investigator, might confound the trial results

### Enrolment, randomisation and retention

Interested participants will complete the informed consent process with a member of the research team. This involves full written and verbal presentations of the study information, with all participants providing written, informed consent prior to commencement of formal study screening and eligibility confirmation. For potential participants aged under 18 years, a parent or legal guardian will be included in each stage of this process. Details of complaints and compensation procedures, and contact information for research personnel are included in the participant informed consent form. In addition to study consent, participants will be asked if they consent to their data being used for future research, and if they consent to being contacted for future research opportunities. Participants can opt in or out of one or both of these and still participate in INVEST.

Once eligibility for the trial is confirmed, the trial coordinator will randomly and consecutively assign participants to receive 39 weeks of either IPS or UVS, in a ratio of 1:1, using a password-protected computer programme, with a randomisation sequence that is computer-generated. Randomisation uses permuted blocks and is stratified by gender (female/not female) and age (< 18 years/18–25 years).

A graduate research assistant (RA) will conduct initial screening and will determine eligibility (in consultation with the study coordinator and principal investigator). The RA will also conduct all research assessments at 13, 26, 39 and 52 weeks. Intervention group assignment will be concealed from the RA (single-blind) conducting the assessments and from the biostatistician conducting the primary analyses. Blinding of the RA will be achieved and maintained by frequently reminding clinicians of the importance of the blind, excluding the RA from all clinical discussions, and forbidding the RA from accessing participants’ medical records. Any cases of un-blinding will be documented and another ‘blind’ RA will conduct assessments thereafter. Blinding of the RAs will be tested at the end of their employment or the end of the study, using an online self-report instrument designed specifically for the study. Study investigators will not be blind to group allocation, and the nature of the intervention precludes concealment from participants or clinicians.

Recruitment and retention in the trial will be maximised with thorough training of study RAs, including study-specific and GCP training. A comprehensive participant-tracking database will be utilised, and research assessments will take place on site at Orygen, at the participant’s home, or in the community, according to participant preference. Whether or not an individual chooses to participate in the trial will have no bearing on the clinical care that they receive in HYPE.

### Interventions

All participants will receive treatment in the HYPE programme at Orygen. This has been published elsewhere [49] and is comprised of general psychiatric care, clinical case management, and up to 16 sessions of individual psychotherapy where indicated. The programme is delivered within an overall relational framework (Relational Clinical Care), derived from CAT. Treatment received (i.e., number of sessions, case management v CAT) will be captured for each participant.

### IPS

IPS is a client-driven intervention that will be delivered by a qualified and fully trained IPS practitioner. The IPS practitioner identifies the employment and/or education goals of each individual and provides ongoing support to the young person, even following engagement in appropriate employment/education. IPS practitioners also provide support (education, advice, etc.) to workplaces and educational institutions, in order to facilitate and maintain the engagement of the young person. IPS was developed as an evidence-based employment services model for people with severe mental illness [51]. Since IPS was originally developed for adults with mental illness, a modified version of the intervention will be used. Modified IPS models, to include education, have been implemented successfully overseas [43, 44, 47], and within Orygen [52]. The model has eight core principles:

1. *Competitive employment and mainstream education*. The main aims of IPS are to help job seekers find employment in the open labour market and to help clients with educational goals find suitable educational programmes in mainstream institutions. IPS practitioners do not help clients find unpaid internships or sheltered employment
2. *Eligibility based on client choice*. Involvement in an IPS service is based on a desire to achieve vocational goals in a competitive job or mainstream education. Clients are not excluded on the basis of diagnosis, disability or symptoms
3. *Integration of vocational services and mental health services*. IPS providers and mental health treatment teams are closely integrated and work collaboratively
4. *Attention to client preferences*. Services are based on individual client preferences. IPS practitioners actively approach employers and educational institutions based on the young person’s career preferences rather than referring to mismatched opportunities that employers need filled. Decisions regarding education and employment are based on client strengths, interests, and what they may have done in the past and previously enjoyed
5. *Personalised benefits counselling*. IPS practitioners provide or help find accurate and understandable information about the effects that work will have on the job seeker’s welfare payments
6. *Rapid job search*. IPS providers start looking for employment opportunities immediately rather than providing lengthy assessments, training and counselling. IPS practitioners work with clients in the first session to develop a vocational plan based on client preferences, past experiences, interests, skills and strengths
7. *Targeted job development*. IPS practitioners build employment and educational provider networks and relationships through systematic contacts guided by client preferences. IPS practitioners form relationships by developing an understanding of each business and its human resource needs, explaining to hiring personnel what IPS is, and the benefits for participating businesses/institutions
8. *Ongoing individualised support*. IPS clients can continue to receive support for as long as they require. IPS practitioners continue ongoing support long after a placement is found. Frequent contacts between employment specialists and clients are important to help with any required training and difficulties faced in the new environment

In the current RCT, ongoing individualised support from the IPS practitioner (principle 8) will cease at 39 weeks from baseline. The IPS practitioner will work with each participant to develop an individualised support plan, which might include case managers, family members or other service providers. This deviation from the model is necessary to maintain IPS practitioner caseload and study recruitment at acceptable levels. Time-limited IPS is comparable to traditional IPS in terms of employment outcomes [53], and 6 months of IPS has proven successful in assisting young people with first-episode psychosis to find employment [54] and to engage with education [52].

Adherence to the IPS model will be assessed using the IPS fidelity scale [55], which will determine the extent to which the IPS intervention is delivered according to the prescribed model. INVEST will utilise a modified version of the IPS fidelity scale designed to capture both educational and employment information, specifically for young people, ensuring a realistic measure of model adherence with the study population.

Each participant randomised to IPS will be assigned an IPS practitioner and will receive IPS services in accordance with the IPS fidelity scale. The un-blind study coordinator will provide relevant participant information to the IPS practitioner following randomisation and facilitate contact between specialist and participant. The IPS practitioner will meet with the participant as soon as possible, within 14 days following randomisation, to establish the participant’s short- and long-term vocational goals. Suitable employment/education opportunities will be identified, after which the IPS practitioner will provide support and advice regarding applications, writing a curriculum vitae, preparing for interview, and accompanying clients to interviews when this is the client’s preference. Sessions and contact with the IPS practitioner will vary in terms of length and communication method, but ongoing support will be provided as required or requested by the participant. Method of communication (e.g. telephone, face to face), length of interaction, and topics discussed will be recorded for each interaction between the IPS practitioner and participant. A participant will be considered discontinued from the intervention in the event of five failed contact attempts within a 1-month period or if a participant chooses to cease their involvement with the intervention. In the event of discontinuation, participants will still be permitted to provide research data if desired.

### UVS

UVS reflects the vocational support that is routinely available via case managers for young people attending the HYPE programme at Orygen. This includes the following educational support, offered in conjunction with Travancore School – a Victorian Department of Education and Training school providing education to those aged 15–18 years, while engaged with mental health services:

- *Work it out (pathways planning).* A young person meets with a Travancore teacher and their case manager to discuss vocational goals and provide career pathway planning and support
- *Secondary consultation*. A Travancore teacher provides educational information and advice to the young person’s case manager, and family where appropriate. A secondary consultation does not include any school visits or educational setting liaison
- *Outreach*. A Travancore teacher offers outreach support to young people in a current education setting, or transitioning to a new setting
- *School’s in*. Learning support is provided within a small-group setting to a young person who wishes to re-engage with education

Employment support is not offered as part of UVS at Orygen. Case managers inform young people that this type of support can be obtained through Centrelink and/or employment agencies, and refer to such agencies. Centrelink is the Australian Government Department of Human Services programme that delivers payments and services for the unemployed, people with disabilities, students and apprentices. HYPE case managers have no specialised training or expertise in education networks, employment seeking or workplace support.

### Outcome measures at the primary endpoint

#### Primary outcome

The primary endpoint of the trial is 52 weeks after baseline assessment. Assessments will also occur at 13, 26 and 39 weeks after baseline.

The primary outcome is the number of days spent in mainstream education and/or employment since baseline. Because some study participants might engage in both education and employment during the course of the intervention, the number of days spent in each of education and employment will be separately recorded. To capture these data as accurately as possible, participants will receive the following two text-messages each week:

1. ‘Orygen research. Think about the past 7 days, not including today. On how many days did you attend work or employment for which you were paid?’
2. ‘Orygen research. Think about the past 7 days, not including today. On how many days did you attend secondary school, education or training, working towards a qualification (for example VCE, VCAL, Certificate, Diploma, Degree, etc)?’

Participants will reply to each message with a number only (0–7). In order to compensate for any missed messages/non-responses, participants will be asked at each research assessment how many days they have attended education/employment in the past 13 weeks. The mean, minimum and maximum of the total numbers from the text-messages and the research assessments will all be considered in the analysis.

#### Secondary outcomes

1. Cost-effectiveness of IPS compared with UVS. Intervention costs will be determined using study budgetary information, as well as the client contact information maintained by IPS practitioners. Other costs will be measured using a Resource Use Questionnaire (RUQ) adapted for use in this study. This questionnaire includes information on primary and specialist health and mental health care, hospitalisation, pharmaceuticals and other health and non-health sector services (such as accommodation), as well as other non-health sector resource use, such as other vocational services. Medicare (MBS) and the Pharmaceutical Benefits Scheme (PBS) administrative data will also be requested for participants who consent to the collection of these data. Welfare benefits as a primary source of income, and new commencements on welfare benefits, will be measured at each time point
2. Quality of life, as measured by the AQoL-8D (Assessment of Quality of Life) [56]. This 35-item self-report questionnaire assesses for quality of life across eight dimensions: Independent living, Pain, Senses, Mental Health, Happiness, Coping, Relationships, Self-worth. It can also be used to determine quality-adjusted life years (QALYs) for economic analysis of the intervention
3. BPD severity, as measured by BPDSI-IV (Borderline Personality Disorder Severity Index) [57]. This 70-item semi-structured interview is based upon the SCID-II BPD module. It assesses current frequency and severity of each *DSM-IV* BPD criterion

### Additional measures

#### Interview/researcher rated

- The SCID 5-RV (Structured Clinical Interview for *DSM-5* Research Version) [58] will be used to assess for mental-state pathology and for syndromal diagnoses
- The SCID 5-PD (Structured Clinical Interview for *DSM-5* Personality Disorders) [58] will be used to assess for personality disorder pathology and for syndromal diagnoses
- The SOFAS (Social and Occupational Functioning Assessment Scale) [59] is a single-item scale (1–100) used to rate social and occupational functioning

#### Self-report

- The BSL-23 (Borderline Symptom List-23) [60] is a 23-item measure that assesses the severity of BPD pathology, and is sensitive to change over time
- The CEF (Comprehensive Evaluation of Functioning) is a 92-item measure designed to assess for functioning across four domains: social, education and employment, self-care and health, and participation in society. This measure was designed specifically for use in young people across the study age range (Fowler et al., in preparation)
- The MFDU (Monitoring the Future, Drug USe) [61] scale is a 26-item measure to determine short-term and lifetime drug use
- The PID5-BF (Personality Inventory for *DSM-5* brief form) [62] is a 25-item measure of the 5 *DSM-5* Section III personality trait domains: negative affect, detachment, antagonism, disinhibition and psychoticism
- The SDS (Sheehan Disability Scale) [63] assesses for disability and functional impairment and is sensitive to change
- The SMU (Social Media Use Questionnaire) is an eight-question measure that is used to determine social media use [64].
- The SRASBM (Self-report of Aggression and Social Behaviour Measure) [63, 65] is a 56-item measure that assesses for six relationship behaviours: Relational Aggression, Physical Aggression, Relational Victimisation, Physical Victimisation, Exclusivity, and Prosocial Behaviour

The assessment schedule is detailed in Table 1.

**Table 1:** Schedule of assessments

	Screening	Time point (weeks)				
		Baseline (0)	13	26	39	52
Demographics	X		X	X	X	X
BPDSI-IV		X			X	X
RUQ briefer baseline economic evaluation		X				
RUQ full-version			X	X	X	X
SCID-5 RV module A	X				X	X
SCID-5-RV modules B, C, D, F, G, I, K, L		X			X	X
SCID-5 PD BPD module	X					
SCID-5-PD other modules		X				
SRASBM		X				
AQoL-8D		X	X	X	X	X
BSL-23		X	X	X	X	X
PID-5 brief version		X		X		X
SDS		X		X		X
CEF		X	X	X		
MFDU		X		X		X
SMU			X			

*AQoL-8D* Assessment of Quality of Life, *BPDSI-IV* Borderline Personality Disorder Severity Index, *BSL-23* Borderline Symptom List-23, *CEF* Comprehensive Evaluation of Functioning, *MFDU* Monitoring the Future Drug Use, *PID-5* Personality Inventory for DSM-5 brief form, *RUQ* Resource Use Questionnaire, *SCID 5-PD* Structured Clinical Interview for DSM-5 Personality Disorders, *SCID 5-RV* Structured Clinical Interview for DSM-5 Research Version, *SDS* Sheehan Disability Scale, *SMU* Social Media Use Questionnaire, *SRASBM* Self-Report of Aggression and Social Behaviour Measure

### Data management

Trial data will be safely stored in password-protected computer databases, accessible only by members of the research team. Paper copies of participant files will be stored in locked filing cabinets in a swipe access storage area, co-located in a monitored building with research personnel. All participant files will be de-identified, with each participant allocated a unique identification number. Personally identifiable material, such as study consent forms, will be stored separately from research data files. This is fully explained in the participant informed consent form.

Data integrity will be supported by a range of means. The trial database is programmed to have range checks for data values, minimising the chance of data being recorded incorrectly. Data verification, through double data entry, will be undertaken on a random sample of 30 cases at baseline, with an a priori acceptable data-entry error rate set at 0.5%. More data-checking will be conducted if the error rate is above 0.5%. Data monitoring will be undertaken by the trial sponsor for the duration of the study. The monitor will be available to advise and assist with issues relating to study progress, protocol, regulatory and ethical adherence, and data accuracy.

### Statistical methods

Study sample size and power calculation are based on comparing groups in terms of the primary outcome measure. A sample size of 54 per group (*N* = 108) should detect an effect size of 0.62 with 80% power and a 5% significance level. This allows for a 14% dropout rate, based upon two previous RCTs conducted in the same population and setting [66, 67].

The main intent-to-treat data analysis will employ a *t* test to compare the two intervention groups in terms of the primary outcome measure. As a secondary analysis, the randomisation stratification variables of age and gender will be used as covariates and the two intervention groups will be compared using the general linear model. Other covariates, such as educational level and employment history, will also be considered. Logistic regression and general linear model analysis will be employed to analyse the secondary outcome measures, depending upon the nature of the measures. Multiple imputation will be considered if the amount of missing values is non-trivial.

The cost-effectiveness of IPS, compared with UVS, will be determined by a cost-consequences analysis comparing the intervention with comparator, with the full spectrum of differences in outcomes included in the study.

### Dissemination of results

Results of the study will be presented in publications, presentations and any other forum in a de-identified form, at the group-analysis level. Study results will be made available on the Orygen website, presented in plain language. Authorship of publications will be in accordance with the Orygen publication policy, agreed by study investigators.

## Discussion

Currently, specialised psychosocial treatment for BPD achieves significant symptomatic recovery but it does not necessarily lead to functional improvement. Moreover, functional impairment appears to be persistent and to have lifelong consequences for individuals with subthreshold borderline pathology or BPD, their families and society.

INVEST addresses this issue by asking whether adding IPS, a specific vocational support intervention, to specialised, evidence-based psychosocial treatment for BPD, achieves superior outcomes for young people, compared with UVS. INVEST represents the first RCT of IPS for individuals with a primary diagnosis other than a psychotic disorder, and the first RCT of IPS for people of any age group with borderline pathology. INVEST will also integrate educational and employment interventions to meet the developmental needs of young people during the peak period of onset for severe mental disorders.

The INVEST RCT has several strengths. In order to reflect ‘real world’ clinical practice, there are minimal exclusion criteria. Participants with concurrent mental disorders, including bipolar, psychotic, substance use or antisocial personality disorders, who are usually excluded from RCTs in BPD, are free to participate in the trial. The inclusion of people with a psychotic disorder diagnosis is justified, as IPS is already a proven intervention for individuals with such diagnoses. However, previous trials have not routinely measured borderline pathology among these participants. This heterogeneous group of INVEST participants (with a common feature of borderline personality pathology) will be highly representative of young people presenting to a frontline specialist youth mental health service, increasing the external validity of the trial.

To date, most RCTs conducted with people with BPD have restricted their age range to either teenagers or ‘adults’ (18 years and older). The age range for INVEST will be 15 to 25 years, which recognises the distinct and developmentally coherent period in economically developed societies, extending from puberty to around 25 years of age, which is believed to support the acquisition of the culturally embodied knowledge, skills and self-regulatory capacities that are needed to achieve independent adult-role functioning and integration into society [68, 69]. Broadening the age range for inclusion in INVEST increases the capacity of the intervention to address both education and employment, along with the crucial issue of transition from school to post-school education or employment. Orygen commences offering services at age 15 years, increasing the opportunity for early detection of individuals with borderline pathology and of those with vocational impairment. INVEST explicitly targets young people with ‘early stage’ BPD and it will recruit young people with a broad range of BPD severity (3–9 BPD features). While the main objective of this RCT is to evaluate the overall effectiveness of IPS, compared with UVS, among young people with borderline pathology, should IPS prove effective, it will be possible to investigate BPD severity as a moderator of treatment response.

Each participant will be well-characterised, having undergone a comprehensive, standardised, multi-method assessment. This assessment will include characterising the stage of illness, including whether previous evidence-based BPD treatment has been received. It is expected that most participants will be presenting for their first episode of evidence-based treatment for borderline pathology. Such early detection minimises course-of-illness effects, such as iatrogenic harm, concurrent disorders and treatment effects.

Care has been taken to minimise confounding of the study findings. For example, randomisation will be stratified by age and gender, and all participants will receive the HYPE intervention as background treatment, which is manualised and standardised. The selection of an active, characterised comparison condition, including the use of government programmes, such as Travancore School’s Work it Out and government JobActive services, is superior to the use of either a waiting list or uncharacterised treatment as usual condition. The IPS intervention is manualised and written guidelines detail the nature of UVS, supporting the potential replication of these interventions, should they prove effective. Treatment fidelity will be determined using standardised scales.

While the trial design has attempted to address many problems identified in the literature, some limitations to this RCT are anticipated. For practical reasons, INVEST will not be able to recruit 12–14 year-olds, limiting its external validity with regard to very young people with BPD. All INVEST participants will be offered the comprehensive HYPE intervention. It is possible that there might be a ‘ceiling effect’ of this high-quality intervention for improvement in the primary and secondary outcomes. However, previous data indicate that vocational outcomes in HYPE lag behind those for psychopathology (hence, the rationale for the study). All participants will continue to draw upon usual vocational services throughout their participation in the trial. While the routinely available educational support at Orygen, via the Travancore School, might reduce the effect size for IPS with regard to educational outcomes, it is not expected to affect employment outcomes [46]. It was considered unethical and impractical to deny INVEST participants access to this ‘usual care’, which has been available for many years, during the trial. Importantly, uptake of this educational support is highly variable among the population attending HYPE. Actual use of all vocational supports will be captured at each research assessment, enabling the results to be interpreted with this in mind.

The transition from childhood to adulthood is the critical period for educational achievement, learning vocational skills, and early employment experience and training [70]. However, this is also the peak period for the onset of severe mental health difficulties, which can greatly disrupt these processes. Successful implementation of IPS in an early intervention service for BPD, evidenced by improved engagement in education and employment, is expected to provide greater functional improvement for young people with BPD. Successful engagement in vocational pursuits profoundly affects the lives of individuals with mental ill-health, with resulting improvements in finances, self-esteem and self-worth, and reduced stigmatisation [51]. In turn, this is likely to lead to economic benefits, such as greater productivity, reduced dependency upon welfare payments, and less mental health service usage. Such outcomes would have benefits for individuals, families and society.

### Trial status

The trial was registered (ACTRN12619001220156) on 3 September 2019. The trial is recruiting, with the first participant enrolled on 2 October 2019. Recruitment is expected to be ongoing until 30 June 2021. This protocol is version 4, dated August 2019.

## Data Availability

The datasets used and/or analysed during the current study are available from the corresponding author on reasonable request.

## Abbreviations

AQoL-8D: Assessment of Quality of Life
BPD: Borderline personality disorder
BPDSI-IV: Borderline Personality Disorder Severity Index
BSL-23: Borderline Symptom List-23
CEF: Comprehensive Evaluation of Functioning
HYPE: Helping Young People Early
GCP: Good Clinical Practice
INVEST: INdividual Vocational and Educational Support Trial
IPS: Individual Placement and Support
MFDU: Monitoring the Future, Drug USe
NEET: Not in education, employment or training
PID-BF: Personality Inventory for *DSM-5* brief form
QALYs: Quality-adjusted life years
RCT: Randomised controlled trial
RUQ: Resource Use Questionnaire
SCID 5-PD: Structured Clinical Interview for *DSM-5* Personality Disorders
SCID 5-RV: Structured Clinical Interview for *DSM-5* Research Version
SDS: Sheehan Disability Scale
SMU: Social Media Use Questionnaire
SOFAS: Social and Occupational Functioning Assessment Scale
SRASBM: Self-Report of Aggression and Social Behaviour Measure
UVS: Usual vocational services
